# Systematic Comparison of Genetic Algorithm and Basin Hopping Approaches to the Global Optimization of Si(111) Surface Reconstructions

**DOI:** 10.1021/acs.jpca.2c00647

**Published:** 2022-05-06

**Authors:** Maximilian N. Bauer, Matt I. J. Probert, Chiara Panosetti

**Affiliations:** † Department of Physics, University of York, York YO10 5DD, United Kingdom; ‡ Technical University of Munich, Lichtenbergstraße 4, 85748 Garching, Germany; § Fritz Haber Institute of the Max Planck Society, Faradayweg 4, 14195 Berlin, Germany

## Abstract

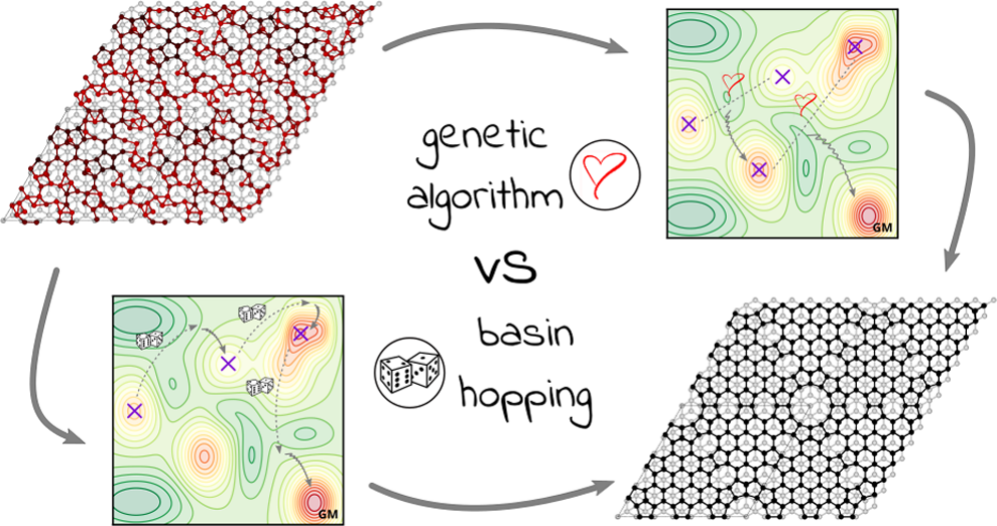

We present a systematic study of two widely used material structure prediction methods, the Genetic Algorithm and Basin Hopping approaches to global optimization, in a search for the 3 × 3, 5 × 5, and 7 × 7 reconstructions of the Si(111) surface. The Si(111) 7 × 7 reconstruction is the largest and most complex surface reconstruction known, and finding it is a very exacting test for global optimization methods. In this paper, we introduce a modification to previous Genetic Algorithm work on structure search for periodic systems, to allow the efficient search for surface reconstructions, and present a rigorous study of the effect of the different parameters of the algorithm. We also perform a detailed comparison with the recently improved Basin Hopping algorithm using Delocalized Internal Coordinates. Both algorithms succeeded in either resolving the 3 × 3, 5 × 5, and 7 × 7 DAS surface reconstructions or getting “sufficiently close”, i.e., identifying structures that only differ for the positions of a few atoms as well as thermally accessible structures within *k*_B_*T*/unit area of the global minimum, with *T* = 300 K. Overall, the Genetic Algorithm is more robust with respect to parameter choice and in success rate, while the Basin Hopping method occasionally exhibits some advantages in speed of convergence. In line with previous studies, the results confirm that robustness, success, and speed of convergence of either approach are strongly influenced by how much the trial moves tend to preserve favorable bonding patterns once these appear.

## Introduction

**Figure 1:**
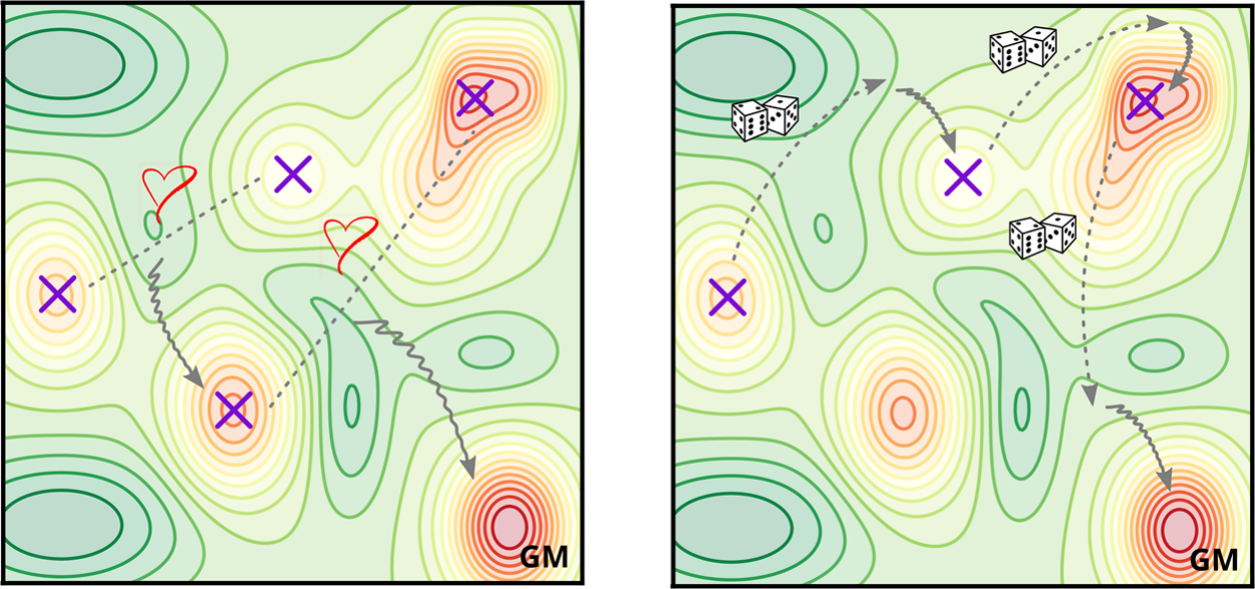
Schematic representation of how Genetic Algorithms (GA, left) and Basin Hopping (BH, right) explore the potential energy surface (color online). Left: two (or more) local minima geometries (represented by crosses) are allowed to mate (heart) to produce two (or more) offspring geometries that are relaxed to new local minima (wiggly arrow). If the offspring survives, it is in turn allowed to mate in the next generation. Right: a local minimum geometry is randomly perturbed (dice) and relaxed (wiggly arrow) into a new local minimum. If accepted, the new local minimum is perturbed in the next step. If successful, both procedures, repeated, will converge to the global minimum (GM).

Finding the lowest energy of a molecule or crystalline structure by means of unbiased global optimization is a fascinating, prominent challenge in computational materials modeling. As one may expect for a problem that has, in general, no guaranteed solution, there is correspondingly no “foolproof” approach to solve it. Thus, not surprisingly, a plethora of different global optimization algorithms flourished over the last two decades, from simple stochastic schemes as simulated annealing^1^ and ab initio random structure search (AIRSS)^2,3^ to sophisticated heuristics such as landscape paving,^4^ particle swarm optimization,^5^ cascade genetic algorithms with multistep refinement of the target quantity,^6^ or neural-network controlled dynamic evolutionary approaches.^7^ Among all, two popular families of global geometry optimization techniques include Monte-Carlo-based (“physics-” or “maths-inspired”) methods, such as Basin Hopping (BH),^8^ and heuristic, evolutionary principles-based (“biology-inspired”) Genetic Algorithms (GA).^9^ No general rule for preferring a specific algorithm has been identified, as the efficiency of classical global optimization methods is both property- and system-dependent.^10^ Recently, the global optimization challenge is finding invaluable support in the employment of artificial intelligence, in combination with standard techniques. Surrogate energy models based on machine learning can be incorporated to accelerate the global search, often with the global screening itself concomitantly used to train the model.^11−13^ Clustering allows exploration of the configurational space efficiently,^14,15^ and Bayesian statistics can be used to tune the balance between exploration and exploitation.^16^ Active learning is increasingly popular in conjunction with both stochastic^17^ and evolutionary^18^ methods. Taking one step even further ahead, the identification of plausible atomistic structures, especially for materials discovery, can bypass the “classical” exploration of the potential energy surface altogether, by means of reinforcement learning^19^ and deep-learning generative models.^20^ We note in passing that, complementarily, global optimization approaches can in turn aid the generation of machine-learning atomistic potentials, by providing a physically motivated protocol to hierarchically refine training sets. Naturally, machine learning approaches can benefit from the “energy landscape” perspective, as discussed, for example, in ref (21). In the following, we will focus exclusively on the “standard” GA and BH algorithms. A schematic representation of the two basic algorithms is depicted in Figure 1.

GAs have been proven successful in finding periodic organic and inorganic crystal structures. For example, the GA of the electronic structure package CASTEP has been tested successfully on periodic, highly symmetric crystal structures and polymorphs.^22^ Four surface reconstructions of rutile TiO_2_ (all in accordance with experiments) have been found by the Genetic Algorithm in the USPEX program^23^ The Birmingham parallel genetic algorithm (BPGA) introduced in 2003 identified the most stable configuration of Iridium clusters of the sizes *N* = 10 to *N* = 20 atoms^24^ In 2018 GAtor, a Python GA code coupled with structure clustering via the machine learning module Scikit-learn has been presented and tested on molecular crystals.^25^ In 2019 a GA was designed which performs simultaneously an optimization of the crystal and the magnetic structure.^26^

BH enjoys widespread success thanks to its simplicity, unbiased character, and the need for only a few parameters. The standard BH approach has proven highly reliable for the optimization of clusters and biomolecules.^27^ A further development was proposed in 2004 with the Basin Hopping with occasional jumping (BHOJ), which introduces random jumping processes without rejection to mitigate stagnation.^28^ The BH algorithm showed efficiency and robustness also in the identification of all-atom protein foldings.^29^ In 2010 a BH Algorithm was presented^30^ which was tweaked by optimizing the escape steps such that the initial atomic and cell velocities are aligned to low-curvature directions of the current local minimum. So-called generalized BH approaches exploit the quasi-combinatorial nature of the potential energy landscapes of multicomponent systems, by defining bi- or multiminima in more than one metric space. These approaches have been successfully applied to nanoalloys,^31−33^ as well as to the combined structure and sequence optimization of proteins.^34^ It has been recently shown^35,36^ that the efficiency of BH in finding relevant low-lying structures in covalent systems is significantly improved by employing trial moves based on Delocalized Internal Coordinates (DICs) that tend to preserve favorable structural motifs throughout the simulation. BH can also effectively resolve surface structures of complex adsorbates,^37^ especially in conjunction with Bayesian frameworks.^38^

Global optimization techniques for material structure prediction inherently require a very large number of energy and force evaluations, no matter how efficiently they are designed, which to date has limited their application to either small system sizes using accurate first-principles methods, or large system sizes using more approximate methods. As a result of the computational cost, global optimization of large extended systems, such as those of interest in materials science and surface science, is relatively scarce. Only recently have global optimization methods been used on lower-dimensional structures such as interfaces and grain boundaries,^39^ 2D spherical topologies,^40,41^ and adsorbates on surfaces.^36,37,42−44^ There are even fewer systematic studies of the effect of algorithm parameter choice on efficiency. A most notable effort is presented in ref (45); however, to the best of our knowledge, no systematic investigation has been performed specifically for surface science and materials science. This work is a systematic study of global optimization for what is possibly the most complex surface structure known—the Si(111)-(7 × 7) reconstruction. While the structure and energetics of this reconstruction are known, the rationale for studying it here is that this is a tough test of a surface reconstruction prediction algorithm—can the full Si(111)-(7 × 7) reconstruction emerge from an unbiased structure search method? Hence, the insights gained from this study should be relevant to many (simpler) systems. The Si(111)-(7 × 7) reconstruction is known to be described by the so-called Dimer-Adatom-Stacking fault (DAS) model^46^ which was finally derived after many years of joint effort by experiments and theory. Every 7 × 7 supercell contains 12 adatoms, a corner-hole, 8-rings, and double 5-rings as structural motifs. The number of removed or additional atoms Δ*N* with respect to the unreconstructed *N* × *N* surface supercell (including the smaller and simpler 3 × 3 and 5 × 5 reconstructions which also follow the DAS model) is given by
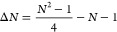
1

However, while the structural details of the 7 × 7 reconstruction of a Si(111) surface are now known, “predicting” it from first-principles calculations has been a major challenge in computational surface science. The difficulties arise as one needs a very large unit cell to contain the elaborate symmetry (at least 49 atoms in each layer) and many layers to contain both the surface reconstruction and the relaxation with depth to the bulk structure. While a pioneering DFT calculation by Payne et al.^47^ of the 7 × 7 reconstruction showed that it was stable and lower energy than the unreconstructed surface (which was taken as an ab initio proof of the accuracy of the DAS model), the input structure was taken as the DAS model. The Payne et al. study also showed that the 7 × 7 reconstruction was more stable than 5 × 5 and 3 × 3, but they did not perform a global optimization or “predict” the DAS structure. In this paper, a series of global optimization searches were performed to see if (and under what search conditions) the DAS reconstruction emerges as the global minimum.

## Theory

In this study, the performance of the Genetic Algorithm (GA) and Basin Hopping (BH) approaches in finding the global minimum energy structure—the DAS surface reconstruction—are assessed, and the effect of key algorithm parameters is systematically investigated. In the algorithms employed here, the only additional feature with respect to the most basic recipe is the implementation of “smart” trial moves, namely, the DIC displacements mentioned above for BH, and a cut-and-splice procedure tailored to surface reconstructions for GA. In principle, feature-preserving ideas based on DIC displacements can also be implemented in GA approaches, by, e.g., introducing mutations in DICs. It has to be noted here that the cut-and-splice procedure already facilitates the passage of favorable structural motifs to the offspring, as entire groups of atoms are preserved intact in the children structures at each generation.

For completeness, we note that the concept of smarter trial moves had also been previously introduced in the form of symmetrization schemes in ref (45). Those have been proven to perform equally well with both BH and GA.

### Genetic Algorithm

A Genetic Algorithm (GA) is a very general approach to global optimization and can be used to optimize many different kinds of problems. It is based on Darwin’s idea of “survival of the fittest”^49^ and was translated by Holland into an algorithm that mimics natural selection^50^ to solve the global optimization problem. The first implementation for materials structure prediction was for molecules^9^ and then extended to periodic systems^22^ to study crystals. A full description of the periodic system implementation is available in ref (48). The key idea is to have a population of *N* structures that are available for “breeding” and to evolve toward the global optimum solution over a number of generations. The periodic lattice vectors and the fractional coordinates of the ions are used to represent the structure (“DNA”); a periodic cut(s) is used to breed two parent structures to make two children (“crossover”) and then allow for various random mutations (such as displacement, swaps, etc.) in the new child structures. Once the child structures have been generated, they then undergo a local relaxation, and the final relaxed energy is used to rank according to their fitness. To be precise, genetic algorithms that involve a minimization step of the members after each generation are regarded to be “Lamarckian” rather than “Darwinian”, as discussed in refs (51, 52). The optimization step is analogous to the local optimization after each step in the basin hopping approach. As such, GA with local optimization steps is expected to exhibit comparable performance with BH. Fitness is a function of the appropriate free energy (usually the enthalpy), and fitter structures have lower free energy. If the number of atoms in the children is allowed to vary (a “grand canonical” search), then it is more appropriate to use the Gibbs free energy per atom as the input to the fitness function. Finally, the fitness is used to reduce the 2*N* structures (parents and children) to *N* to make the set of parents for the next generation. Fitness is also used to select pairs of structures to act as parents, from which the next generation may be bred.

In this study of elemental silicon, the only mutation considered is a random displacement of any atom after breeding, which has a “mutation amplitude” and occurs randomly according to a prescribed “mutation rate”. It has been found that a significantly larger mutation is advantageous in generating the initial population of generation 0 so as to get a broad sampling of configuration space—the “initial ion amplitude” (IIA). In global optimization, it is important to explore as much as possible of the configuration space (or equivalently, the potential energy surface (PES)) as well as to find the best candidate for the global minimum. This is a challenge, and no global optimization approach can *guarantee* that the global minimum will be found, only that the longer the run, the larger the chance of finding it. Normally, if the best structure found has not been improved upon for a long time (and ideally across multiple runs starting from different initial conditions), then it is considered a good candidate for the global minimum. Factors such as mutation rate, mutation amplitude, and size of the population can all affect the ability of the algorithm to find the global minimum.

If the drive to the global minimum is too aggressive, then there is a risk of stagnation, where all of the members of a population become too similar, and the ability to search for new areas of configuration space is lost. The risk of stagnation can be significantly reduced by measuring the similarity of different structures in a population to the lowest-energy structure found at that point, and penalizing structures that are too similar.^53^ This encourages a wide range of structures to be maintained in the population. The overall fitness of a structure *f*_*i*_ is then a weighted combination of two different fitness measures: *f* _*i*_^energy^ based upon minimizing the free energy, and *f* _*i*_^diss^ based upon maximizing the structural dissimilarity
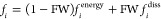
2where FW ∈ [0,1] is the “fitness weight”. This is clearly not the only possible strategy to mitigate stagnation in global optimization algorithms. For example, niching^54^ has been used in other GA algorithms and will be investigated in follow-up studies. A different approach to balance exploration (probing unknown regions) and exploitation (finding lower-energy structures) is to design a GA with a Bayesian acquisition function as a fitness function.^16^ This latter work showed that the search for molecular compounds could be made much faster, but crystalline surface reconstructions were not found to be significantly faster than with a non-Bayesian search.

**Figure 2:**
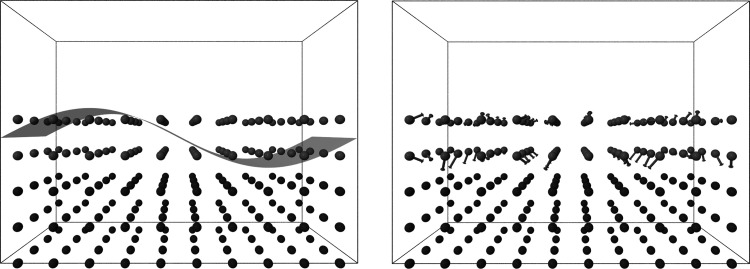
Schematic representation of (left) the surface cut procedure^48^ employed in the GA mating and (right) the Delocalized Internal Coordinates displacement employed in the BH throughout this work. Left: a sinusoidal cut propagates groups of contiguous atoms together from parents to children, facilitating the preservation of favorable bonding patterns. Right: gray arrows represent global displacements in DICs. DIC displacements are concerted motions of groups of atoms, thus also facilitating the preservation of favorable bonding patterns. In both panels, dark gray atoms represent the bulk layers, which are not modified during the mating or the trial moves but allowed to relax. Light gray atoms belong to the “active” layers, which are modified by the mating or the trial moves.

The appropriate free energy can be the enthalpy (for a single element, fixed number of atoms with variable volume calculation), the enthalpy/atom (for a single element, variable number of atoms calculation), the Gibbs free energy/atom (for a multielement, variable number of atoms calculation), or others. The GA crossover operation results in two children with the same total number of atoms as in the two parents, but can also be constrained to ensure that both children have an equal number of atoms. This flexibility can be useful in performing variable stoichiometry searches but is not needed here. The GA of Probert and Abraham^22,48,53^ was adapted to be appropriate for finding surface reconstructions. Instead of having two periodic cuts in the bulk, this study uses just one periodic cut that goes through the surface layer(s) as shown in Figure 2 (left). This surface cut has a random wavelength (but commensurate with the box) and a random amplitude, with a maximum amplitude of 1/10 of the height of the cell. In this way, the bulk of the structure is not affected, but surface structural motifs can propagate between generations, and atoms can be added or removed from different parts of the surface.

### Basin Hopping

Basin Hopping samples the configuration space through consecutive jumps from one local minimum of the potential energy surface to another. The jumps are achieved by randomly moving one or more or all atoms in the system to new positions. Each move is followed by a local geometry optimization. Acceptance or rejection of the thus-created structure proceeds on some appropriate criterion—most commonly, the Metropolis criterion;^55^ however, different acceptance schemes have been proposed, including threshold acceptance^56^ and Tsallis statistics.^57^ In this work, displacements were performed in Delocalized Internal Coordinates (DICs) which have been shown to enable more efficient exploration of the configuration space for covalent systems^35,36^ than traditional moves in Cartesian coordinates (CCs). The DICs are nonredundant linear combinations of internal coordinates, obtained by singular value decomposition (SVD) of the transformation matrix between Cartesian coordinates and internal coordinates. The latter are defined as a redundant set automatically obtained by detecting bonded atoms if separated by a distance equal to the covalent radius of the species considered multiplied by a tolerance factor and subsequently extracting the angles between them. For the details of the coordinate transformation, the reader is referred to refs (35, 36). The DICs resulting from the SVD constitute a nonredundant, complete set of collective displacement directions (modes). At each BH iteration, a pre-set number of DIC modes is randomly drawn to construct the displacement for the global trial move. The “chemically sensible” trial moves generated as such mostly consist of concerted motions of groups of atoms, which preserve favorable bonding patterns and drive the random walk toward more relevant regions of the configuration space than Cartesian trial moves, yielding a preferential sampling of lower-energy regions of the PES. Additionally, the generated trial geometries tend to be less strained (reducing the number of relaxation steps to the corresponding local minimum) and less prone to relax to dissociated structures. Here specifically, the surface-adapted DIC definition is used (Complete Delocalized Internal Coordinates, CDIC, hereafter interchangeably referred to as CDIC or DIC) as described in refs (35, 36). In the latter, the system is partitioned into two (or more) subsystems. Each subsystem is assigned its own set of coordinates, the concatenation of which recovers a full-dimensional coordinate vector that is equivalent to Cartesian coordinates. The system is partitioned into bulk and surface layers and the DIC displacement is applied to the surface layers only, as shown in Figure 2 (right). In our definition of CDICs, translations and rotations (which are naturally filtered out by the construction of DICs), are re-included as separate displacement modes that can be picked up in the random selection to construct the total displacement. This is useful when sampling the adsorption of molecules on surfaces,^36^ but not necessarily in the context of sampling surface reconstructions; therefore, in this work, translational and rotational displacements were turned off. The cost function to minimize can be generally defined as the formation energy of the surface reconstruction, as directly derived from ref (58). This allows, in principle, to take into account the variation of the number of particles naturally as the corresponding Metropolis criterion will be a function of the chemical potential, like in grand canonical and semi-grand canonical BH approaches proposed before.^59^ Alternatively, one may minimize the free energy directly.^60^ Throughout this work, however, the number of atoms per structure was kept constant and the different (known) compositions sampled separately in the canonical ensemble.

Finally, as noted in the Genetic Algorithm section, stagnation can be a problem in any global optimization algorithm. For example, with BH the already mentioned in ref (45) makes use of a “taboo list”, while rejection-free moves, such as in BHOJ,^28^ may also be implemented. Neither of these approaches were used in this study.

### Comparison

GA and BH explore the configuration space in a profoundly different manner. While the GA evaluates a number of structures (equal to the chosen population size) in parallel for each generation, BH is sequential as it evaluates only one structure per iteration. To clarify, we refer to BH as “sequential” in its standard formulation. While parallel formulations of BH exist, that exploit replicas in a parallel tempering manner (e.g., ref (61)), those are not considered here. Therefore, one may expect that a comparable exploration of the configuration space is achieved for a number of BH steps equal to roughly population size times the number of generations in the GA. To compare the two global optimization methods taking into account this difference, a normalized measure of efficiency η is used, which was introduced in ref (62) for the purpose of comparing different global optimization algorithms
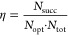
3where *N*_succ_ is the number of structures that are within some acceptance window (see below) of the global minimum (if known), *N*_opt_ is the number of structures evaluated before the global minimum of the whole run is found, and *N*_tot_ is the total number of structures evaluated in the entire run. For this test system, the global minimum—the DAS structure—is already known and hence it is straightforward to see if the global minimum (GM) has been found. More generally, the GM is not known a priori and so may be taken as being the best candidate structure found during (all) the run(s) so far. Furthermore, the interest is not just in finding the global minimum, but all structures which are thermally accessible, i.e., are within some appropriately chosen convergence window of the global minimum. For simple bulk structures, an appropriate choice would be *k*_B_*T* per atom, or in the case of the surface reconstructions considered here, *k*_B_*T* per surface area. Defined as such, η is simultaneously a measure of “how fast” (through *N*_opt_) and “how well” (through *N*_succ_) the algorithm explores the configuration space.

Another key metric for comparing different algorithms and parameter choices is robustness *R*, defined as the fraction of attempted calculations that converged to the GM—or, more generously, to within the appropriate thermally accessible energy of the GM. In this work, the stricter criterion is used. Only runs that find at least one structure within the convergence window of the global minimum (*N*_succ_ ≠ 0) were included in the efficiency calculation. Runs that fail to meet this criterion (hence η = 0) contribute to reducing the robustness *R* instead.

Runs that are within the thermally accessible range but fail to reach the GM have *N*_opt_ = *N*_tot_. As such, this will slightly overestimate the efficiency, assuming that at best the run would have identified the GM in the next step. However, these runs also contribute to reducing the robustness *R* despite having η ≠ 0 (and not necessarily small). A good algorithm will therefore have high efficiency and high robustness. Different algorithms, or different parameter choices for an algorithm, will affect its location in this efficiency-robustness space, and an optimal approach will lie on the Pareto front in this space (cf. Supporting Information). In the GA approach used here, structures are assigned to generations and a whole generation is optimized before fitness is assigned and breeding takes place for the next generation. Hence, *N*_opt_ is calculated as the number of members in a generation (i.e., the population size) times the number of generations evaluated, up to and including the generation in which the GM is found. An alternative GA approach, based upon breeding from a pool of structures and continuously updating the fitness and parents without the generational step would therefore be slightly more efficient. For BH, *N*_opt_ is simply the iteration at which the GM is first identified, *N*_succ_ is the number of structures in the entire run that fall within the convergence window of the GM, and *N*_tot_ is the total number of iterations. Additionally, it is useful to define one or more measures of stagnation *S*. One may consider, e.g., the number of “stuck” runs where the best energy found has not improved for a number of iterations; additionally, if the global minimum is known, one may partition the latter into “converged” (i.e., stuck to the GM), or “stuck to suboptimal”. However, for both stochastic and evolutionary searches, any chosen metric is somewhat arbitrary. First, there is no direct way to evaluate how much of the configuration space has been sampled, nor a guarantee that something in the next step will or will not drive the simulation to a new local minimum. For evolutionary methods, one may identify stagnation either as the point at which the entire population converges to a certain solution (quite conclusive, less the effect of mutations), or if the fittest member has not changed for a certain number of generations (and, clearly, how many is completely arbitrary). For stochastic methods, there is no direct equivalent to the “flat” population convergence of the GA, but one may still identify stagnation if, for example, no new local minimum is accepted, or no significant gains in energy are achieved for a certain (again, arbitrary) number of steps. In the following, stagnation *S* is defined as the fraction of “stuck” runs (regardless of whether converged to the GM or not). For GA, a simulation is considered to be stuck if the average enthalpy of the current population is within *k*_B_*T*/unit area from the enthalpy of its fittest member, and/or if no new fittest member is identified in a preconvergence window of  generations. In the latter case, the GA algorithm stops the search automatically. Of note, the two criteria do not necessarily coincide; therefore, the stagnation number for GA is the fraction of runs that meet at least one of those criteria. For BH, a simulation is stuck if no gains in energy larger than *k*_B_*T*/atom have appeared for more than 50% of the total steps. For this purpose, we assume a temperature of 300 K.

## Computational Details

All calculations were performed applying periodic boundary conditions. The initial structure for each simulation contained a slab of six layers of silicon atoms, of which the bottom three layers were constrained to the bulk coordinates and the top three layers are allowed to be optimized in any direction. A vacuum layer of 10 Å is added above the surface to avoid interactions with the periodic image. To enable an exhaustive study of the different parameter combinations, which would be prohibitive at the DFT level for the targeted system sizes, energy and forces were evaluated with the surface-modified^63^ Stillinger–Weber model potential.^64^ The latter does not require the addition of a hydrogen passivation layer. Local relaxations were performed using the BFGS algorithm^65−68^ as implemented in the electronic structure code CASTEP(69) for the GA and in the python package Atomic Simulation Environment (ASE)^70^ for the BH.

The GA was performed using a maximum of 40 generations and 40 members for all runs of the system size 3 × 3, 60 generations and 60 members for 5 × 5, and 150 generations times 150 members 7 × 7. To base the performance assessment of the GA on a sufficient set of statistics to derive meaningful statements, 10 runs with different random number seeds were completed for each set of parameters. Structures in generation zero for the GA runs were obtained by displacing the reconstructed surface by the chosen initial ion amplitude (IIA). The number of atoms was kept fixed in accordance with eq 1 for all children. The deployed Genetic Algorithm is implemented in a developers’ version of the electronic structure code CASTEP(71) and therefore not yet available to the public; its release is planned for early 2022. A sinusoidal surface cut of amplitude 3 Å was centered about the initial surface layer so that only the two uppermost layers were effectively involved in the reconstruction. The third layer was allowed to relax together with the top 2, while the three bottom layers mimicking the bulk were kept fixed.

For strict comparability, 10 BH runs for each reconstruction size and each parameter combination were performed starting from 10 initial structures drawn randomly from structures created in the GA at generation zero with an initial ion amplitude (IIA) of 1.4 Å. As BH has fewer parameters to control, a full screening was performed varying both the step size *dr* between 1.00, 1.20, 1.40, and 1.75 Å and, for each of those, the number of DICs employed to construct the global displacement between one single DIC, 25, 50 and 75% of the maximum number of available modes. For each step size, BH runs with Cartesian displacement were additionally performed for comparison. All of the BH runs were performed at a pseudo-temperature of 300 K. The effect of the temperature was not investigated here, as the focus of the work is primarily on the effect of the strictly geometric parameters. Similarly to the GA mating protocol, in the BH, the DIC displacements were applied to the two uppermost layers. The third layer was allowed to relax, while the bottom three bulk layers were kept fixed. To evaluate a number of structures comparable to the number of structures evaluated in the corresponding GA run, the number of steps was set to 1600, 3600, and 20 000 for 3 × 3, 5 × 5, and 7 × 7, respectively. Of note, an exact correspondence is not strictly necessary when using the normalized efficiency metrics defined in eq 3; however, it is sensible and fair to compare simulations with numbers of evaluations of the same order of magnitude. The BH approach employed here is implemented in the freely available python package winak.(35,36)

## Results and Discussion

### 3 × 3 Reconstruction

In the following, we present an extensive discussion on the effect of the various parameters that can be tuned in GA and BH searches. While one may generally choose them within sensible ranges, a systematic screening can give insights into best practices to tune the search toward desired outcomes, especially in the global structure optimization of complex systems, where one is typically not only interested in the GM, but more generally in a wider range of thermally accessible configurations. To some extent, many of the involved parameters provide some form of control of the exploration–exploitation trade-off. For example, introducing more frequent large-amplitude mutations in GA, or employing a larger step size in BH, will push the simulations more toward unexplored regions of the potential energy surface. Similarly, one may tune up exploration by directly modifying the cost function in GA (via the fitness weight) or accepting more unfavorable structures (via the temperature) in BH. Conversely, choices in the opposite direction will certainly lead to a solution faster—however, the risk of stagnation to a suboptimal solution increases concomitantly. Even though a thorough screening of the effect of different parameters was only performed for the 3 × 3 reconstruction, we expect similar trends to apply to the 5 × 5 and 7 × 7 reconstruction and, indeed, to any other structure search.

#### GA Results

##### Initial Ion Amplitude (IIA)

To test the ability of the GA to converge to the GM even from strongly distorted structures, the initial random displacement (IIA) was increased from 1.2 to 1.9 Å in steps of 0.1 Å. Table 1 shows the average efficiency η̅, robustness *R*, and stagnation *S* (as defined in the Comparison section). There were 10 independent repetitions for each parameter set, and this was also used to calculate the average generation  and average member  at which the GM was found is noted along with the range of efficiency from η_min_ and η_max_.

**Table 1:** Effect of Varying the Initial Ion Amplitude IIA on the GA Performance for the 3 × 3 Surface^a^

IIA (Å)	1.2	1.3	1.4	1.5	1.6	1.7	1.8	1.9
η̅/10^–5^	148.6	87.8	74.8	31.6	22.2	13.7	9.8	3.9
*R*	1.0	1.0	1.0	0.8	0.9	0.6	0.5	0.2
*S*	1.0	1.0	0.9	0.6	0.3	0.3	0.1	0.1
	2.1	4.0	4.8	9.0	11.3	15.5	14.8	19.0
	84	160	192	360	453	620	592	760
η_min_/10^–5^	44.6	24.5	18.8	4.4	4.3	2.5	1.7	0.1
η_max_/10^–5^	237.7	234.9	226.5	103.8	74.9	71.2	27.0	13.1

a All calculations were for a population size of 40 members per generation, and a maximum of 40 generations, and repeated 10 times. In addition to the average efficiency η̅, robustness *R*, and stagnation *S*, results for the average GA generation  and member  at which the global minimum is identified and the range of efficiency from η_min_ and η_max_ are reported. Note that values of η are quite small, typically 10^–5^, hence the notation used in the table. The other GA parameters were FW = 0.5, MA = 0.2 Å, and MR = 0.1.

Not surprisingly, larger initial ionic displacements result in increasingly lower efficiency, as generations evolving from very strained, high-energy initial populations take longer to enter the *k*_B_*T* window and, subsequently, the GM will appear later. This in turn can lower the robustness (for runs with a fixed number of generations), as some runs will not converge to the GM at all, unless the maximum number of generations is increased to compensate for the more exhaustive search.

We additionally observed that, for small IIAs, a number of GA runs found the GM in the initial population. In general, GA runs with too small IIA are likely to stagnate very soon—if not to the GM, then to some local minimum in the super-basin spanned by the IIA as can be seen by the value of *S*. To be clear, determining whether convergence to the GM is “premature” carries a certain degree of arbitrariness.

Finally, we note that a displacement of 1.2 Å equates to ∼50% of the Si–Si bond length, while 1.8 Å is ∼78%. The latter is a situation where the system is completely disordered and yet still the algorithm can find the ordered structure. Not surprisingly, however, the highest efficiencies are observed for IIA values between 1.2 and 1.4 Å, which are less likely to completely break bonds in the initial generation and so choosing IIA = 50% of the average bond length seems to be an efficient and transferable heuristic. This suggests that parameters that facilitate the preservation of bonding patterns are generally favorable.

##### Mutation Rate (MR)

The mutation rate was varied between 0.2 and 0.9 in intervals of 0.1. As shown in Table 2, the effect of this parameter is relatively small. All of the values yield similar performance both in terms of efficiency and robustness. One may identify a weak trend of increasing efficiency with increasing mutation rate; however, the fluctuations are too prominent to consider it significant. The stagnation is also minimally affected.

**Table 2:** Effect of Varying the Mutation Rate (MR) on the GA Performance for the 3 × 3 Surface^a^

mutation rate (Å)	0.1	0.2	0.3	0.4	0.5	0.6	0.7	0.8	0.9
η̅/10^–5^	74.8	74.5	77.6	78.2	73.1	82.8	84.9	80.0	80.5
*R*	1.0	1.0	1.0	1.0	1.0	1.0	1.0	1.0	1.0
*S*	0.9	1.0	1.0	0.9	1.0	0.9	1.0	1.0	1.0
	4.8	4.2	3.8	3.7	4.2	3.7	3.2	3.6	3.6
	192	168	152	148	168	148	128	144	144
η_min_/10^–5^	18.8	26.1	26.6	29.9	31.3	31.2	41.6	35.5	35.7
η_max_/10^–5^	226.5	219.5	214.4	223.0	227.3	219.4	230.2	225.5	228.4

a All calculations were for a population size of 40 members per generation, and a maximum of 40 generations, and repeated 10 times. The other GA parameters were FW = 0.5, IIA = 1.4 Å, and MA = 0.2 Å.

##### Mutation Amplitude (MA)

As shown in Table 3, increasing the mutation amplitude MA, by which the atoms are randomly displaced after breeding two different structures, has a similar, but slightly less pronounced, effect as the IIA. When the MA is increased, the efficiency drops, albeit less dramatically than for variations in the IIA. The robustness (for a fixed maximum number of generations) is almost unaffected, only starting to decrease for MA = 1 Å. Much larger is the effect on stagnation: already a value of MA = 0.5 Å almost completely eliminates the probability to get stuck in a local minimum (here GM) basin.

**Table 3:** Effect of Varying the Mutation Amplitude (MA) on the GA Performance for the 3 × 3 Surface^a^

mutation amplitude (Å)	0.2	0.5	0.75	1
η̅/10^–5^	74.8	68.3	38.6	12.1
*R*	1.0	1.0	1.0	0.8
*S*	0.9	0.5	0.5	0.6
	4.8	4.4	5.5	6.5
	192	176	220	260
η_min_/10^–5^	18.8	32.1	8.2	1.1
η_max_/10^–5^	226.5	225.8	141.3	62.7

a All calculations were for a population size of 40 members per generation, and a maximum of 40 generations, and repeated 10 times. The other GA parameters were FW = 0.5, IIA = 1.4 Å, and MR = 0.1.

##### Fitness Weight (FW)

Varying the fitness weight (FW) has a modest effect on the GA performance, as shown in Table 4. Previous studies on bulk structures^53^ have found a broad plateau of values around FW = 0.5 were optimal. If the FW is low, then the fitness is dominated by the enthalpy contribution but may be prone to stagnation, while a high FW favors structural dissimilarity and generates a broader range of structures but may therefore be slower to converge to the GM. Here, we find that due to the complexity of the search space, a higher FW is favored but that the GA is capable of finding the GM with any reasonable value of FW.

**Table 4:** Effect of Varying the Fitness Weight (FW) on the GA Performance for the 3 × 3 Surface^a^

fitness weight	0.1	0.3	0.5	0.7	0.9
η̅/10^–5^	61.6	61.3	74.8	83.5	81.6
*R*	1.0	1.0	1.0	1.0	1.0
*S*	1.0	0.9	0.9	0.9	1.0
	5.0	5.3	4.8	3.4	3.7
	200	212	192	136	148
η_min_/10^–5^	18.1	17.0	18.8	34.0	37.9
η_max_/10^–5^	219.7	211.3	226.5	216.6	235.1

a All calculations were for a population size of 40 members per generation, and a maximum of 40 generations, and repeated 10 times. The other GA parameters were IIA = 1.4 Å, MA = 0.2 Å, and MR = 0.1.

#### BH Results

Basin Hopping has overall fewer parameters to control, namely, the pseudo-temperature for the Metropolis acceptance (here kept fixed at 300 K throughout), the displacement step size *dr*, and, in the case of DIC-based trial moves, the number of curvilinear modes employed to construct the displacement. The latter is chosen randomly among all of the available DIC modes to construct the global displacement at every BH iteration. We performed runs varying the step size *dr* for values of 1.0, 1.2, 1.4, and 1.75 Å. For each *dr*, the number of delocalized internal modes used to build the displacement was varied between one single mode, 25%, 50%, and 75% of all of the available DIC modes, and for comparison, Cartesian-based BH was also performed. For each parameter combination, 10 different runs were launched starting from 10 different initial structures chosen as described in Computational Details section.

##### Step Size

Table 5 reports the average efficiency η, robustness *R*, and stagnation *S* as a function of the step size *dr* for BH runs where the displacement mode was kept fixed at 25% of the available DICs. It is not straightforward to identify a clear trend for the efficiency, except for the observation that best results are for step sizes between 1.00 and 1.40 Å. A too large step size (1.75 Å) most likely causes the largest fraction of the trial moves to relax to structures outside the *k*_B_*T*/unit area window, thus reducing the efficiency. The low efficiency for 1.20 Å is somewhat surprising, with the latter being the “natural” step size one would expect for this system (about half the bond length of diamond silicon, cf. GA results as well as ref (35)).

**Table 5:** Effect of Varying the DIC Displacement Step Size *dr* on the BH Performance for the 3 × 3 Surface^a^

*dr* (Å)	1.00	1.20	1.40	1.75
η̅/10^–5^	24.7	13.3	18.3	0.5
*R*	0.4	0.2	0.5	0.1
*S*	0.7	0.7	0.9	0.6
	521	167	117	901
η_min_/10^–5^	1.9	1.4	0.3	0.1
η_max_/10^–5^	138.2	66.1	65.6	2.2

a All calculations were for roughly 1600 steps and repeated 10 times. The number of DICs was kept fixed at 25% of all of the available modes.

It is worth noting however how the smaller step sizes present an extremely large spread in efficiency values, as apparent from the values of minimum and maximum efficiency. This is an indication that occasionally the random walk in BH takes a particularly “lucky” (or “unlucky”) path, not particularly dependent on the combination of parameters (provided that those are chosen sensibly among the ones capable of yielding good results). This aspect of BH may well translate into more sluggish statistics than GA when it comes to the convergence of the effect of parameters with respect to the number of repetitions. In this light, the lower efficiency for 1.20 Å, as well as the fuzziness of the trend may be due to insufficient statistics. Further studies with a higher number of repeats might generate better statistics and hence clarify this issue, but the aim of this study is to get an indicative trend and so 10 repetitions were used for all parameter combinations for consistency.

Similarly, a clear trend cannot be identified for the robustness *R*, which only roughly decreases for increasing step size, as would be expected. This rough trend is consistent with the expectation that larger step sizes will favor exploration over exploitation, causing many runs to fail to reach the GM within the predefined number of iterations. Nonetheless, the value for 1.40 Å falls out of trend, also for the stagnation value. This may be an indication that 1.40 Å is an outlier, rather than 1.20 Å. However, no conclusive statement can be made.

Of note, none of the parameter combinations here reaches full robustness (i.e., *R* = 1.0). This is due to the fact that BH is prone to getting stuck in super-basins with local minima, as will be further discussed in the Discussion—General Trends section. Therefore, even for parameter combinations with a high efficiency, the algorithm may (efficiently!) stagnate into suboptimal super-basins, as can be seen with the value of *S*.

##### Percentage of DICs

Table 6 reports the average efficiency η, robustness *R*, and stagnation *S* as a function of the number of DICs employed to construct the global displacement. Similarly to the step size, no clear monotonic trend can be identified for the efficiency. No clear monotonic trend can be identified for the robustness either. Overall, percentages of DICs between 1 and 50% seem to perform comparably, and, consistently with the previous work,^35^ optimal results are obtained with some intermediate percentage of DIC around 25–50%. The marked spike in efficiency for 50% is most likely due to the strong outlier in η_max_. The stagnation decreases steadily with the percentage of DIC.

**Table 6:** Effect of Varying the Different Percentages of DICs (1 Single Randomly Chosen DIC, and 25–75% of the Available DICs), as well as with CC Displacements, for the 3 × 3 System Size^a^

DICs/CC	1	25%	50%	75%	CC
η̅/10^–5^	14.9	18.3	62.9	2.0	0.01
*R*	0.4	0.5	0.5	0.2	0.0
*S*	0.9	0.9	0.8	0.7	0.5
	338	294	117	1024	--
η_min_/10^–5^	0.5	0.3	1.0	0.8	0.01
η_max_/10^–5^	73.3	65.6	426.3	5.9	0.01

a The step size *dr* was kept fixed at 1.40 Å.

As a final remark, we note how the “traditional” BH with Cartesian coordinate (CC) displacements is profoundly inadequate to sample extended surface reconstructions, despite presenting a lower risk of stagnation. Of all 10 runs with *dr* = 1.40 Å, none identified the GM, only one entered the *k*_B_*T*/unit area window and did so with extremely low efficiency. To be fair, two runs in Cartesian coordinates with *dr* = 1.00 Å do identify the GM, with an efficiency of 2.19. However, it is evident that standard BH in Cartesian coordinates is not a viable choice for the global optimization of extended structures such as large surface reconstructions, unless specific strategies are put into place to enhance the sampling with smarter moves (such as the already mentioned approximate continuous symmetry measures).^45^

#### Evolution in Action

**Figure 3:**
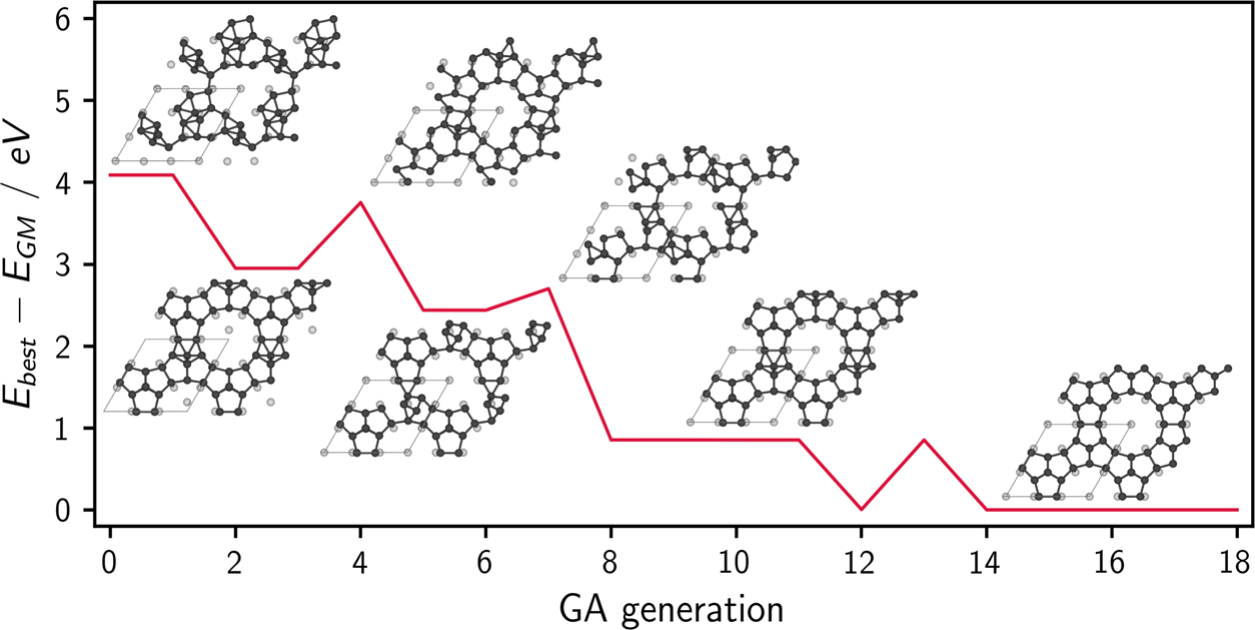
Enthalpy of the best member by generation, with the corresponding geometries overlaid, for a representative GA run with IIA = 1.4 Å, FW = 0.5, MA = 0.2 Å, MR = 0.1, and a population size of 40 members per generation. A clear pattern can be observed: each step in enthalpy corresponds to the appearance of one or more distinct geometric features increasingly similar to those characterizing the DAS reconstruction, such as a corner-hole, dimers, 5-atom rings, etc. Such favorable features are then subsequently passed on to the next generations. A similar trend is observed in DIC-BH runs.

As a visual representation of the global optimization procedure, Figure 3 shows an exemplar GA run for the 3 × 3 surface. We plot the enthalpy of the best member by generation. Of note, since for each generation the member with the lowest enthalpy is automatically allowed to breed, but not directly passed to the next generation, its children may or may not beat it in enthalpy. For this reason, before convergence, the best enthalpy might temporarily increase, as shown by the spikes in Figure 3. This plays an analogous role to accepted uphill moves in BH. The geometries of the best members per generation are overlaid at their corresponding energies. A clear evolution can be observed: each major step downward in enthalpy corresponds to the appearance of one or more distinct geometric features, increasingly similar to those characterizing the DAS reconstructions (corner-hole, dimers, 5-rings, etc.). Such favorable features are then subsequently passed on to the next generations. A similar trend is observed in DIC-BH runs, where the preservation of favorable binding motifs is ensured by the employment of DIC global displacements.

The corner-hole is the first to appear, immediately followed by dimers and 5-rings. Not surprisingly, the last feature to fall into place is the location of one of the two adatoms. In general (especially for the larger 5 × 5 and 7 × 7 reconstructions), we observe a certain difficulty for the global optimization algorithm (more precisely, for the mating in GA and the trial moves in BH) to find the best location for the adatoms. Many candidate solutions above the GM but still within the *k*_B_*T* window have adatoms (or small clusters of adatoms) that are subtly misplaced (see below for more detail). Intuitively, one may argue that such features are not too energetically unfavorable compared to, e.g., ring defects, where the latter generate a larger strain compared to the most favorable ones. Qualitatively, this is compatible with the smaller gains in enthalpy observed for the correct placement of an adatom, with respect to, e.g., the formation of the corner-hole and the rings. The difficulty for a BH displacement to correctly place the adatom is straightforward to understand, as an adatom that is far from where it should be would require a rather improbable displacement to move it to its target position. However, the same difficulty for GA trial moves, which do not suffer from this limitation, can only be explained on the energetic grounds discussed above.

##### Best Structures

**Figure 4:**
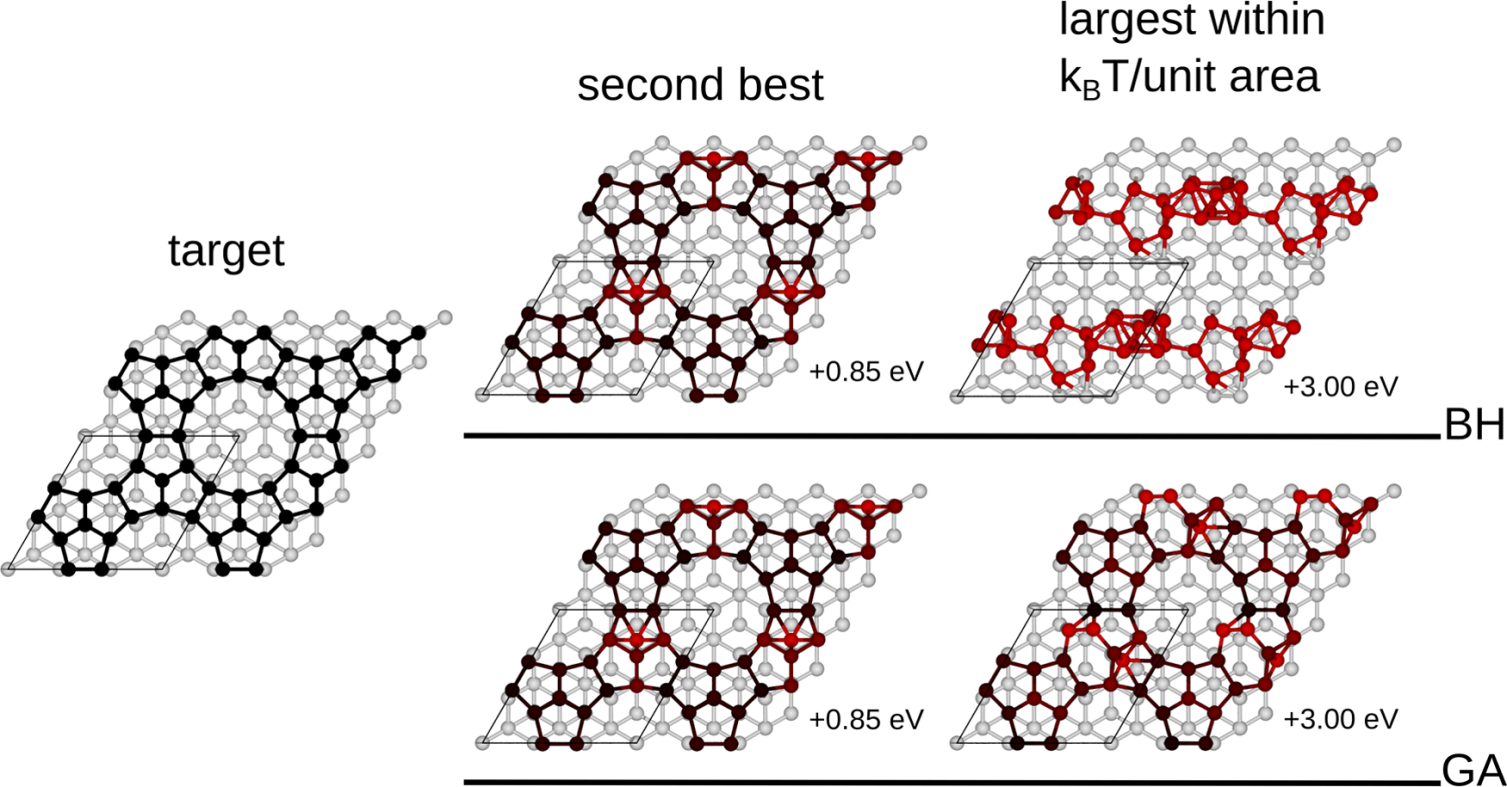
Target structure (left) and representative results from BH (top right) and GA (bottom right) for the 3 × 3 reconstruction. The atoms are color-coded proportionally to the deviation of their positions with respect to the target. As both algorithms succeed in identifying the target solution, here, we show the second-best structure and the highest-energy structure within *k*_B_*T* per unit area. The second-best structure coincides for both methods. Interestingly, the two structures just within the *k*_B_*T* tolerance are profoundly different. The one from GA is much more structurally similar to the target, with just a handful of atoms displaced, while the BH one is very dissimilar, which suggests that completely different alternative reconstructions are, in principle, thermally accessible.

As a final remark, we briefly discuss some candidate solutions found for the global optimization problem by inspecting some representative structures as shown in Figure 4. As both algorithms succeed in identifying the target solution, here we show the second-best structure and also the highest-energy structure within *k*_B_*T* per unit area. The second-best structure coincides for both methods. Interestingly, the two structures just within the *k*_B_*T* tolerance are profoundly different. The one from GA is much more structurally similar to the target, with just a handful of atoms displaced. The one from BH is very dissimilar, which suggests that completely different alternative reconstructions are, in principle, thermally accessible.

### 5 × 5 Reconstruction

The identification of the 5 × 5 surface reconstruction is expected to require many more structure evaluations with either GA or BH. Each structure has 25/9 times more atoms than the 3 × 3 surface reconstruction, and, it is well known (at least in the case of atomic clusters) that the PES has an exponential scaling of the number of minima with the number of atoms.^72^

We therefore fixed the GA parameters at the near-optimal ones found in the earlier study (IIA = 1.4 Å, FW = 0.5, MA = 0.2 Å, MR = 0.1) and increased the population size to 60 members per generation and allowed a maximum of 60 generations per run. Each calculation was again performed 10 times with different random number seeds. With these parameters, 8 of 10 runs found the GM and hence *R* = 0.8 and the efficiency was found to be η = 3.4 × 10^–5^. No runs were deemed to have stagnated by either of the criteria chosen for GA.

For the BH study, we followed the same procedure as before, with initial BH structures drawn from the initial population of the GA run. However, due to the less clear trend in performance wth respect to the parameter combinations seen in the 5 × 5 surface study, we also studied the effect of changing displacement step size *dr* and %DICs for this system size, restricting the screening to step sizes of *dr* = 1.20 and 1.40 Å with 25 and 50% of DICs.

The results are shown in Table 7. These clearly illustrate that the DIC-BH is only weakly dependent on the choice of parameters (as long as the latter are chosen within reasonable boundaries). The parameter combination which was best for the 3 × 3 reconstruction (that is, *dr* = 1.40 Å with 50% of DICs) is not the best for the 5 × 5 reconstruction, and instead, *dr* = 1.20 Å with 25% of DICs is optimal. This is the only parameter set for which DIC-BH found the GM and that in only one run out of 10 repetitions. This parameter combination also produces the best structures closest in average to the solution (cf. Supporting Information), where all 10 of the final results are within the *k*_B_*T*/unit area window. This is in line with the intuitive expectation that a displacement mode preserving the covalent bonding patterns^35^ should generally be preferred.

**Table 7:** Effect of Varying the DIC Displacement Step Size *dr* and %DICs on the BH Performance for the 5 × 5 Surface^a^

*dr* (Å), DIC	1.2, 25%	1.4, 25%	1.2, 50%	1.4, 50%
η̅/10^–5^	2.4	1.7	2.4	2.0
*R*	0.1	0.0	0.0	0.0
*S*	0.5	0.4	0.4	0.4
	1373			
η_min_/10^–5^	0.7	0.2	1.6	0.7
η_max_/10^–5^	6.8	2.3	2.7	2.4

a All calculations were for roughly 3600 steps and repeated 10 times.

**Figure 5:**
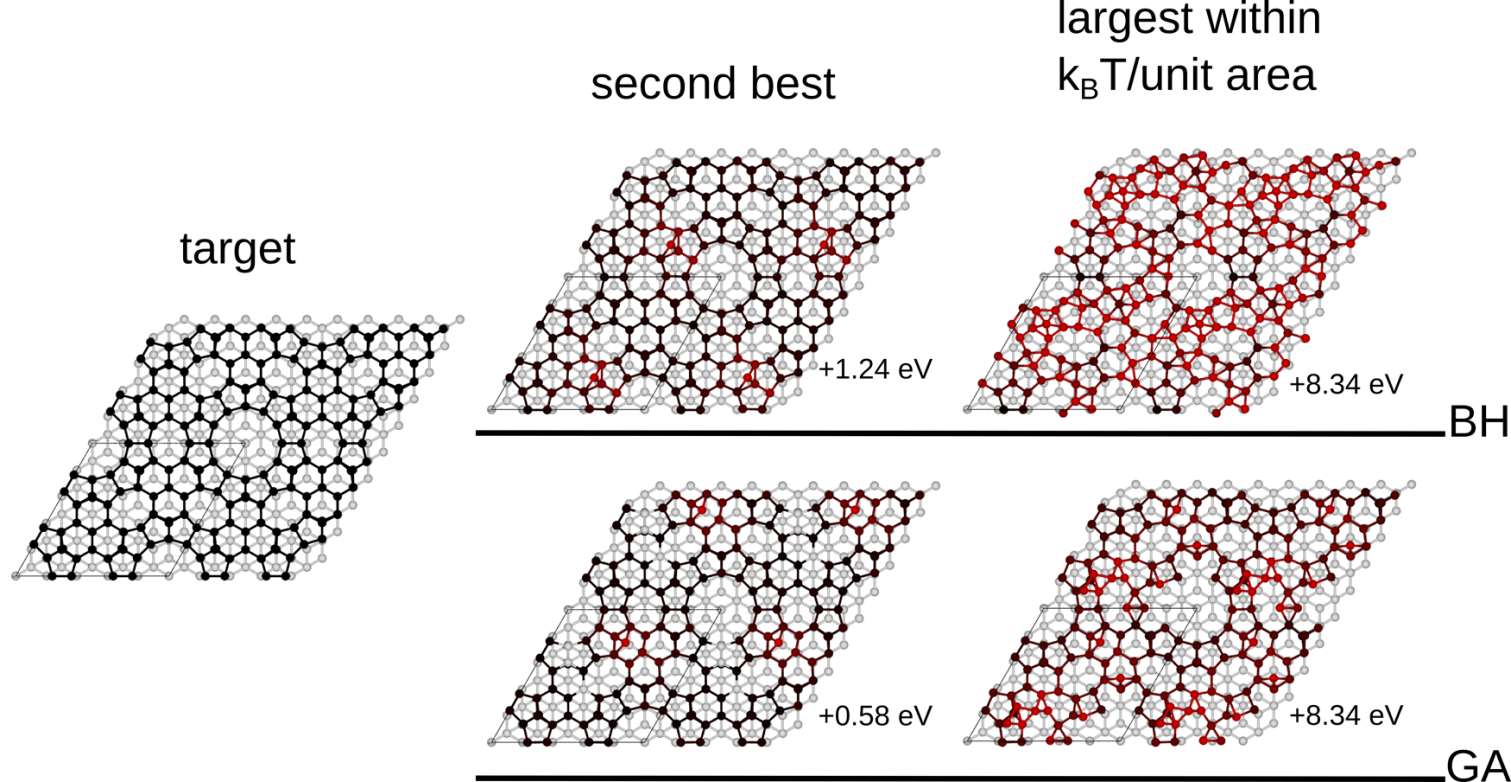
Target structure (left) and representative results from BH (top right) and GA (bottom right) for the 5 × 5 reconstruction. The atoms are color-coded proportionally to the deviation of their positions with respect to the target. As both algorithms succeed in identifying the target solution, here, we show the second-best structure and the highest-energy structure within *k*_B_*T* per unit area. The second-best structure does not coincide for both methods, but both are similar with just one adatom in the wrong place, which, as shown in Figure 3, was the last feature to emerge from the 3 × 3 optimization study as well. Here, the two structures just within the *k*_B_*T* tolerance are much more similar to each other and to the target than those for the 3 × 3 reconstruction.

As for the 3 × 3 reconstruction, we also inspect some representative candidate structures for the 5 × 5 reconstruction as shown in Figure 5. This time, we find structures with similar energy are much more similar; however, the higher-energy one from BH appears more disordered.

### 7 × 7 Reconstruction

The ultimate aim of this study was the 7 × 7 reconstruction. Obviously, this is significantly larger than the 5 × 5 and so the GA was run with a population size of 150 members per generation and a maximum of 150 generations. The GA parameter choices were the same as for the 5 × 5 study. An equivalent number of structures (around 20 000) was also used in the DIC-BH study. Again, 10 runs of each were performed. The results are shown in Table 8, along with the corresponding data for the 3 × 3 and 5 × 5 surfaces.

**Table 8:** Comparison of Efficiencies of GA and Basin Hopping Approaches for Optimal Parameter Sets^a^

	Genetic Algorithm			Basin Hopping		
system	η̅/10^–5^	*R*	*S*	η̅/10^–5^	*R*	*S*
3 × 3	74.8	1.0	0.9	13.3	0.2	0.7
5 × 5	3.4	0.8	0.0	2.4	0.1	0.5
7 × 7	0.16	0.0	0.4	2.1	0.0	0.1

a For GA, the parameters are; MA = 0.2 Å, MR = 0.1, IIA = 1.40 Å, FW = 0.5. For BH, *dr* = 1.20 Å and %DIC = 25%.

**Figure 6:**
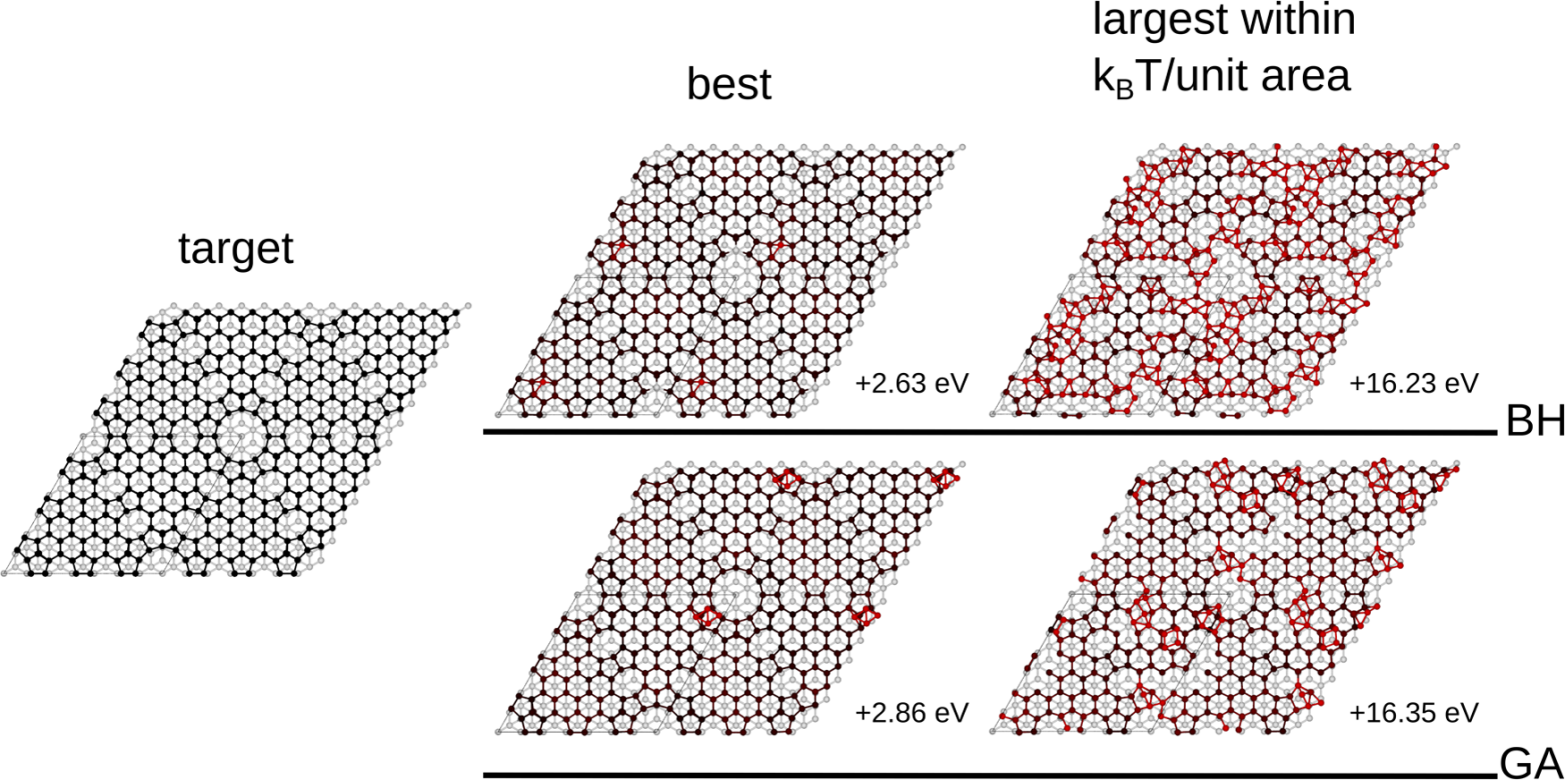
Target structure (left) and representative results from BH (top right) and GA (bottom right) for the 7 × 7 reconstruction. The atoms are color-coded proportionally to the deviation of their positions with respect to the target. As neither algorithm succeed in identifying the target solution, here, we show the best structure and the highest-energy structure within *k*_B_*T* per unit area. The best structure from BH has only one atom displaced, while in the one from GA, some top atoms are grouped in a small Si_4_ cluster. As seen with the 5 × 5 reconstruction, the two structures just within the *k*_B_*T* tolerance are not too structurally dissimilar to the target, but the BH structure shows a higher degree of disorder than the one from GA, despite having essentially the same energy.

Unlike with the smaller system sizes, neither GA nor DIC-BH was able to find the exact DAS reconstruction (GM) for the 7 × 7 structure. Instead, the GA found a structure at 2.9 eV above the GM, corresponding to ∼0.17 *k*_B_*T*/unit area at 300 K, and the best DIC-BH result was 2.7 eV above the GM, corresponding to ∼0.16 *k*_B_*T*/unit area. For GA, no runs stagnated with the criterion of the average enthalpy converging to a certain value for the entire population, while four runs were prematurely ended by the GA algorithm as no new fittest member was identified in the convergence window. As such, the *S* value is 0.4. For BH, one run stagnated, hence *S* = 0.1. Both approaches found good structures that upon inspection are very close to the exact DAS reconstruction, differing only in the position of one atom, as shown in Figure 6. In both cases, the difference from DAS is in the position of an adatom, which as shown in Figure 3, was the last feature to emerge from the 3 × 3 optimization study as well. This suggests that this feature too would emerge here if the runs were made longer.

## Discussion—General Trends

As a final remark, we compare the efficiency and the robustness (with the optimal parameter sets) with respect to system size. Not surprisingly, both efficiency and robustness decrease with system size for both GA and BH, as the complexity of the PES increases. Interestingly, while GA is a clear winner for the 3 × 3 reconstruction, for the 5 × 5 reconstruction, the two approaches show comparable efficiency, while for the 7 × 7 reconstruction, BH becomes more efficient than GA. Conversely, the robustness of GA remains higher than that of BH throughout, and stagnation rates are similar for each system size.

Some qualitative explanation for this behavior may be found in the intrinsic difference in the way the two algorithms traverse the configuration space. In GA, each iteration (generation) uses information from a large portion of configuration space at the same time, as the population is scattered across it (unless it stagnates). As such, it is to be considered a more “global” approach. In a way, with such exhaustive harvesting of information from different regions of the PES, it is reasonable to think that the GM solution would be hard to miss.

In contrast, BH is inherently “locally local”, as its sequential nature (in its standard implementations) only allows new trial moves in the immediate vicinity of the previously accepted minimum, and one at a time. This explains the tendency for BH to get stuck, if the path taken is not particularly favorable, thus lowering robustness. At the same time, when the random walk goes in the right direction, the convergence to the solution is extremely quick. Very often, complex potential energy surfaces exhibit distinct super-basins, which may be significantly separated in terms of configuration distance as seen in disconnectivity graphs.^73^

In the context of Si(111) reconstructions, let us consider the following thought experiment. A certain structure may be very close to the solution, but with a single atom displaced with respect to its correct position by a distance larger than the BH step size (this is the case, for example, of the best 7 × 7 structure found by both GA and BH as shown in Figure 6). As such, it would be impossible for any trial move with that finite step size to exactly place the atom where it should be. This is still possible with multiple moves; however, the path would have to contain intermediate local minima that would need to be accepted. It is then intuitive to understand that, if that is not the case, the two super-basins are disconnected. Conversely, by its nature, the GA would not run into such problems because it would be sufficient that the portion of the structure containing the displaced atom is replaced with a portion with the correct local geometry during mating.

These shortcomings are expected to be alleviated by introducing a similar form of “globality” in BH as well, as it has been proposed in the already mentioned parallel tempering BH.^61^ The latter has been successfully applied to tackle multifunnel potential energy landscapes of disordered proteins,^74^ but, to the best of our knowledge, never in materials science.

## Conclusions

We have performed a systematic study of the effect of parameter choice on the performance of two widely used global optimization algorithms on a challenging test system—the Si(111) surface reconstruction.

It has been shown that both GA and BH approaches are able to find the true global minimum of both the 5 × 5 and 3 × 3 Si(111) surface reconstructions, starting from initial guesses with highly disordered surface layers. Both algorithms struggled to find the exact GM of the 7 × 7 reconstruction, and instead found a nearby structure with only one or few atoms displaced from the recognized DAS reconstruction, and an energy well within the experimentally accessible *k*_*B*_*T*/unit area criterion. We believe that this narrow failure is only due to an insufficiently exhaustive search of the PES and that generating more structures according to the algorithm would fix this, but at a higher computational expense.

We have systematically explored the effect of the different parameters on the performance of the GA and the DIC-BH for this complex system. Our general conclusion is that mutating the initial ionic coordinates in the GA, or analogously displacing in the BH, by IIA = *dr* = 1/2 of the average bond length in the GA is optimal, and this parameter can make a big difference to the efficiency. The other parameters can also make a significant difference to the algorithm performance, and we have evaluated their effect on efficiency, robustness, and stagnation. On the basis of this, we have found a good parameter set for both algorithms that we recommend being used in other studies, without the need for further system-specific optimization.

## Supporting Information Available

Summary of size-dependent properties of the investigated systems and algorithms, visualization of the convergence behavior of the approaches and investigated parameters, and Pareto plots summarizing the overall performance in the *R*–η objective space (PDF)
