# Supplementary material for: Systematic Comparison of Genetic Algorithm and Basin Hopping Approaches to the Global Optimization of Si(111) Surface Reconstructions
Source: J Phys Chem A. 2022 May 6;126(19):3043–56. doi: 10.1021/acs.jpca.2c00647 (PMC9126620; doi:10.1021/acs.jpca.2c00647)
Supplement: Supplementary file 1 — jp2c00647_si_001.pdf [file jp2c00647_si_001.pdf]

Supplementary Information — Systematic Comparison of Genetic Algorithm and Basin Hopping Approaches to the Global Optimization of Si(111) Surface Reconstructions

Maximilian N. Bauer,[†,‡] Matt I. J. Probert,[*,†] and Chiara Panosetti[‡,¶]

†*Department of Physics, University of York, York YO10 5DD, United Kingdom*

‡*Technical University of Munich, Lichtenbergstr. 4, 85748 Garching, Germany*

¶*Fritz Haber Institute of the Max Planck Society, Faradayweg 4, 14195 Berlin, Germany*

E-mail: matt.probert@york.ac.uk

Size-dependent properties

In this study, we consider 3 different related surface reconstructions of Si(111) of increasing complexity and system size. For completeness, Table S1 reports these system dependent parameters, namely: number of atoms N_{atoms} in the simulation supercell, the variation ΔN of the number of surface atoms with respect to the stoichiometric surface, the unit area, the thermal energy tolerance $k_B T$/unit area, and the number of generations defining the preconvergence window in GA.

Table S1

System	$\mathbf{N_{atoms}}$	$\mathbf{\Delta N}$	Unit area (Å^2)	$\mathbf{k_B T}$ / unit area (eV)	GA preconvergence window
3×3	52	-2	116.17	3.00	17
5×5	150	0	322.70	8.34	22
7×7	298	4	632.49	16.35	27

Convergence plots

In the following, we show convergence plots for selected combinations of parameters. Those provide a useful visual aid to the tables in the main text. Solid lines represent the current best structure in a single run, shaded lines represent all the sampled structures, dashed lines represent the average per segment, where a segment corresponds to a generation for GA and to the equivalent number of steps for BH. We also show the thermally accessible energy criterion, viz. $k_B T$/unit area above the GM.

Figure S1 shows the convergence behaviour for GA with respect to variations of the initial ion amplitude (IIA). A clear trend can be identified, *i.e.*, with increasing IIA the convergence to the energy tolerance window is increasingly slower. An IIA of 1.4 Å is to be considered optimal despite showing a slightly slower convergence than 1.2 Å as it maintains a higher degree of genetic diversity whilst at the same time all the runs succeed in finding the GM (unlike with IIA=1.5 Å).

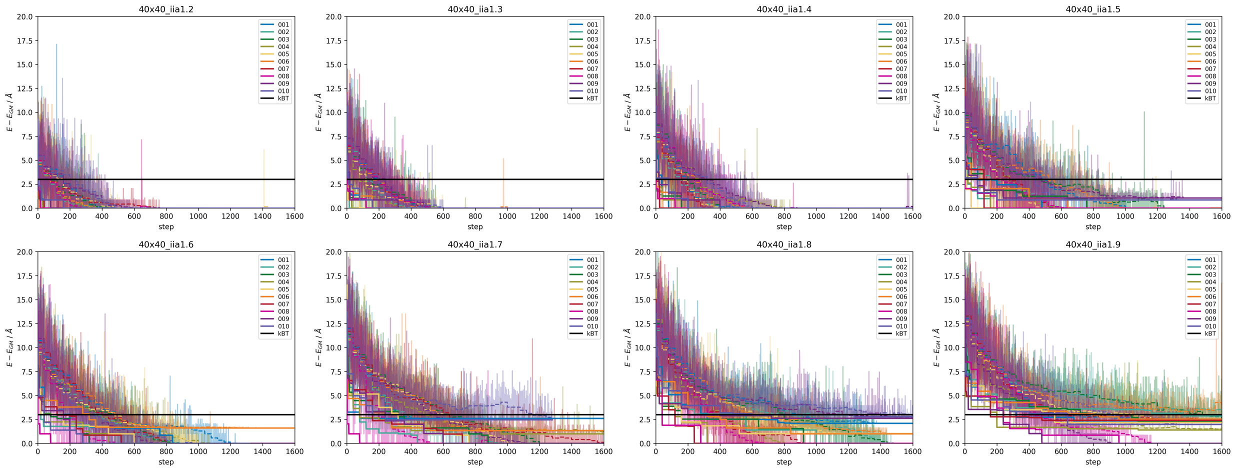

Figure S1

Figure S2 shows the convergence behaviour for GA with respect to variations of the mutation rate (MR). A clear trend cannot be identified here, thus we conclude that the GA is insensitive to this parameter.

Figure S3 shows the convergence behaviour for GA with respect to variations of the mutation amplitude (MA). A clear trend can be identified here, with the convergence speed decreasing dramatically with increasing MA.

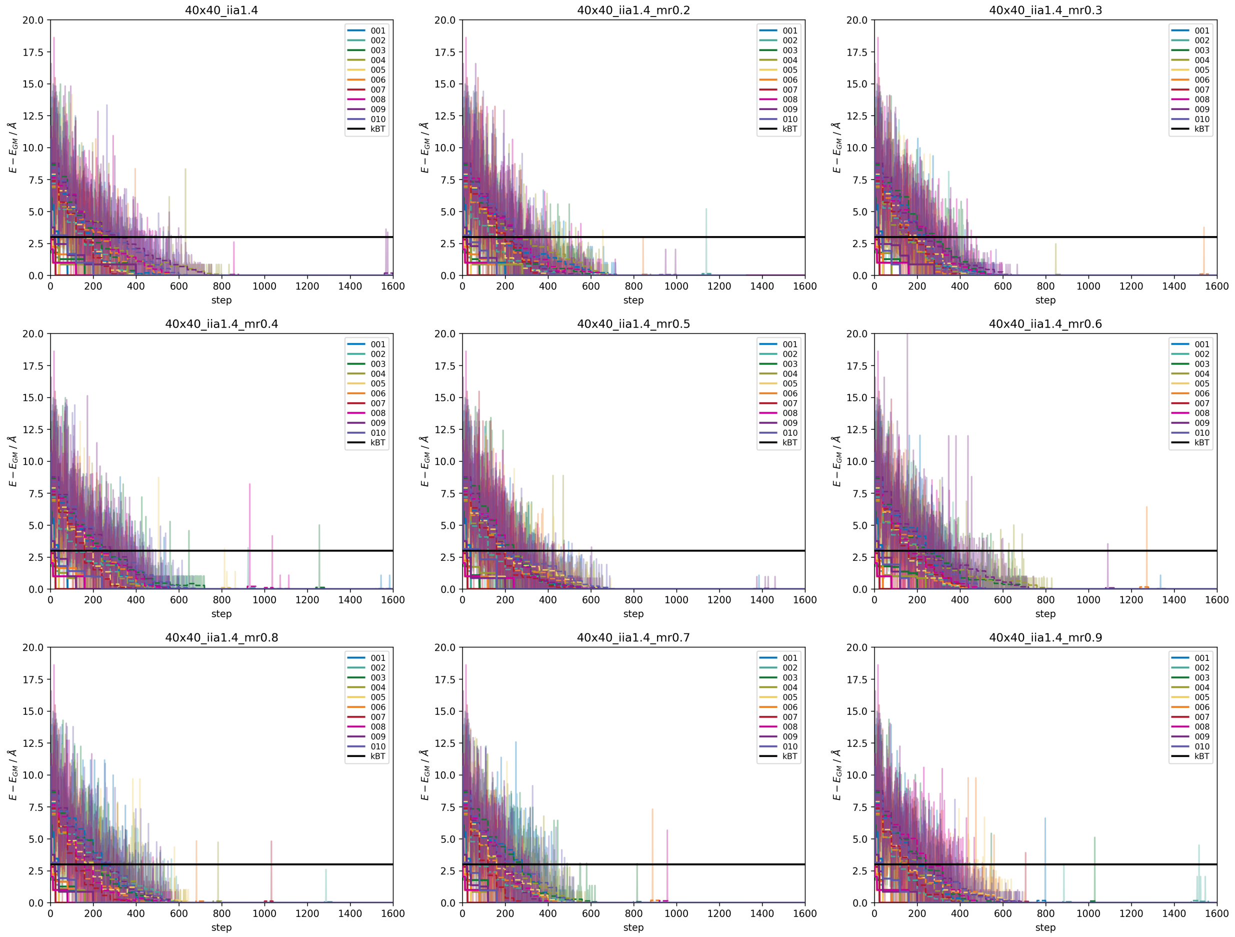

Figure S2

Figure S4 shows the convergence behaviour for GA with respect to variations of the mutation amplitude (FW). Analogously to the MR, a clear trend cannot be identified here, thus we conclude that the GA is insensitive to this parameter.

Figure S5 shows the convergence behaviour for BH with respect to variations of the step size (dr). Analogously to the IIA for GA, the best overall choice is between 1.20 Å and 1.40 Å, as 1.00 Å shows a higher risk of stagnation, while 1.75 Å is too slow.

Figure S6 shows the convergence behaviour for BH with respect to variations of number of DICs, as well as for displacements in Cartesian coordinates (CC). The results appear much less sensitive to this parameter than to the step size. However, the poor performance of CC is evident. Of note, the more DICs are included in the construction of the BH displacement, the more the displacement becomes similar to a CC displacement. This is reflected in the

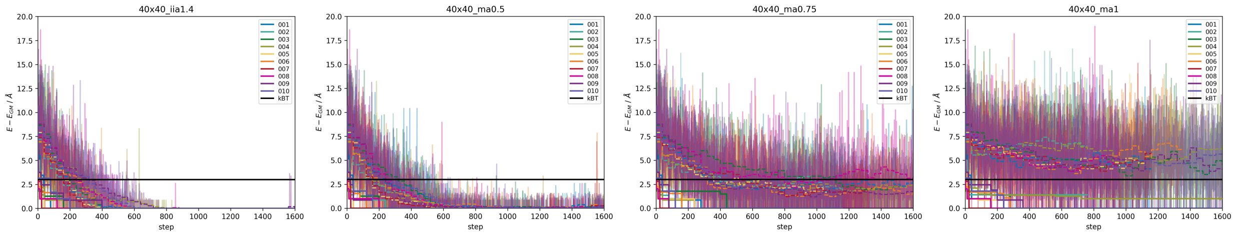

Figure S3

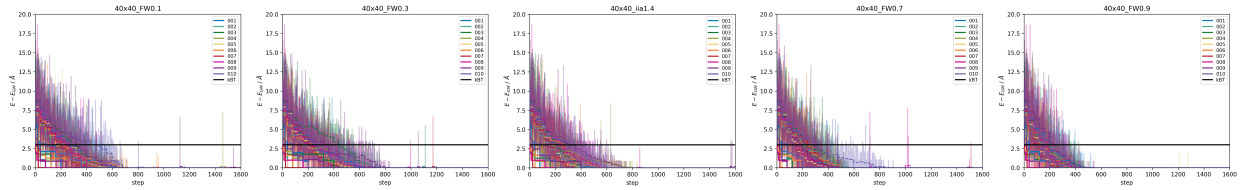

Figure S4

performance for 75 % DIC being more similar to the performance of CC displacements. On the other hand, the opposite limit of only one DIC is more prone to frequent revisits to local minima, thus increasing the risk of stagnation. Therefore, we identify intermediate percentages (here 25 % and 50 %) as optimal.

R-η Pareto plots

In Figure S7, we show R-η Pareto plots for (almost) all the considered parameter sets for both GA (in green) and BH (in black). To avoid excessive crowding of the plots, the values for the varying mutation rate were omitted, as the effect of this parameter is considered minimal (*cf.* main text).

In the left panel, the points in the Pareto plot were calculated, evaluating the robustness in terms of success in finding the exact GM (as in the main text). Here, it is evident that GA perform generally better both in terms of robustness R and efficiency η.

It is also interesting to consider the alternative view of robustness, viz. ability to find a structure within the thermally accessible region instead of the exact GM (right panel). Here, the effect of the individual parameters as well as of the choice between BH and GA becomes

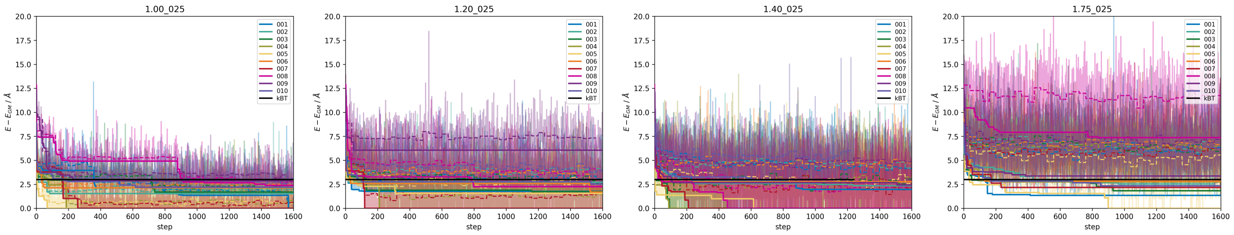

Figure S5

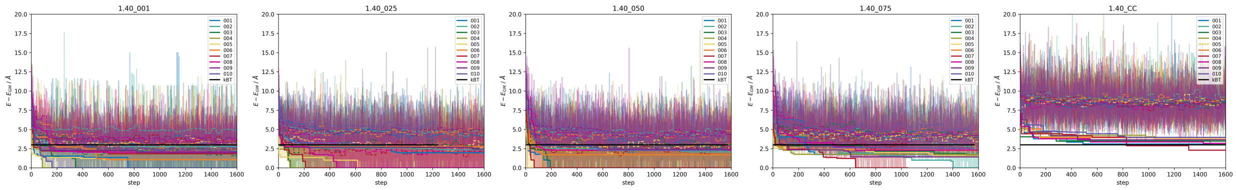

Figure S6

much fuzzier, with even BH in Cartesian coordinates becoming useful for certain parameter choices. We feel the need to point this out, as one cannot conclude that there are necessarily completely "wrong" strategies, as long as one does not trust a single global optimization run and as long as the parameter choices fall within sensible ranges.

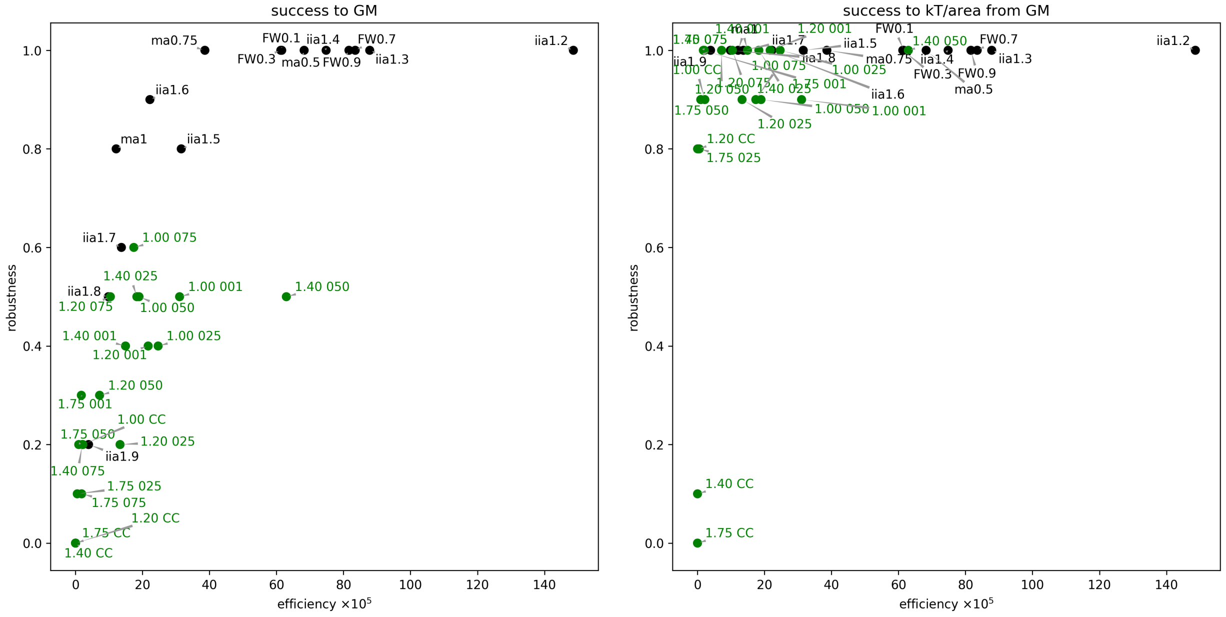
success to GM
success to kT/area from GM
robustness
efficiency ×10^5
ma0.75
FW0.1
iia1.4
FW0.7
iia1.2
FW0.3
ma0.5
FW0.9
iia1.3
iia1.6
ma1
iia1.5
iia1.7
1.00 075
1.40 025
1.00 001
1.40 050
iia1.8
1.20 075
1.00 050
1.40 001
1.00 025
1.20 001
1.20 050
1.75 001
1.00 CC
1.75 050
1.20 025
iia1.9
1.40 075
1.75 025
1.75 075
1.20 CC
1.75 CC
1.40 CC

Figure S7
